# Primary Cueing Tremor: A Task-Specific Tremor of Billiard Players

**DOI:** 10.5334/tohm.1072

**Published:** 2025-08-25

**Authors:** Thananan Thammongkolchai, Lee E. Neilson, Pichet Termsarasab

**Affiliations:** 1 Division of Neurology, Department of Medicine, Faculty of Medicine, Ramathibodi Hospital, Bangkok, Thailand; 2 Department of Neurology, Oregon Health & Science University, Portland, Oregon, USA; 3 Neurology and Research Service, VA Portland Health Care System, Portland, Oregon, USA

**Keywords:** Tremor, task-specific tremor, Task-specificity, sports

## Abstract

**Objective:**

To describe a unique form of task-specific tremor (TST) in billiards players.

**Background:**

Task-specific movement disorders occur during highly learned activities. While task-specific dystonia in billiards players has been reported, TST has not been previously characterized.

**Methods:**

Case series with literature review

**Cases:**

Two right-handed billiards professionals developed tremor specifically during cueing. In Case 1, a 55-year-old experienced right arm tremor triggered by shoulder extension during cue positioning. Tremor resolved upon shooting, improved with alcohol, and was confirmed by electromyography showing a 6.3-Hz tremor. Botulinum toxin provided partial benefit, whereas medications were ineffective. Case 2, a 66-year-old player, had coarse tremor in the non-dominant (left) hand used to form the bridge during his shooting stance, contrasting with Case 1, where the dominant hand holding the cue was affected. Medications were ineffective, but thalamic deep brain stimulation resulted in approximately 70% improvement. Both had subtle bilateral postural and kinetic tremor, and neither showed features of dystonia.

**Conclusion:**

Our cases expand the phenomenology of task-specific movement disorders in billiards players to include tremor. Alcohol responsiveness and electrophysiologic findings suggest a variant of essential tremor. Recognizing primary cueing tremor may have implications for treatment, natural history, and pathophysiology, which likely differ from those of billiards-related dystonia.

## Introduction

Task-specific movement disorders are a distinct category of abnormal movements manifesting exclusively during specific activities. These disorders can include task-specific dystonia and task-specific tremor (TST). Task-specific movement disorders, particularly dystonia, are often associated with repetitive activities, such as writing or playing music or sports, particularly in professional musicians or athletes [1]. Unfortunately, the same intensive practice needed to excel in these activities is a risk factor for developing dystonia, such as various musician’s dystonias (e.g. pianist dystonia, guitarist dystonia, embouchure dystonia) and sports-related dystonia (e.g., the yips and runners’ dystonia) [2].

TST is an action tremor that occurs only when performing or attempting to perform a particular task. TST can similarly affect musicians, manifesting as a primary bowing tremor in bow-string instrument players [3], and can also occur in the general population, presenting as a primary writing tremor or primary smiling tremor [4]. Due to its unclear pathophysiology, there have been controversies regarding whether TST represents a point on the spectrum of focal dystonia or essential tremor (ET) [5]. Both TST and task-specific dystonia have been documented in athletes, albeit infrequently [6,7,8,9,10]. Even more rare are case reports of billiards-related movement disorders; this includes two cases of a professional billiards player who developed focal dystonia when holding the cue [7,10] and one case with positional head “tremor” only when bending the trunk 80–90 degrees in preparation for striking the cue [9]. Herein, we describe two cases of billiard players in whom arm tremor appears exclusively in certain positions while playing. Electrophysiologic studies were also performed in one of these patients, providing insights into the pathophysiology and implications for treatment.

## Result: Case series

### Case 1

**Figure 1:**
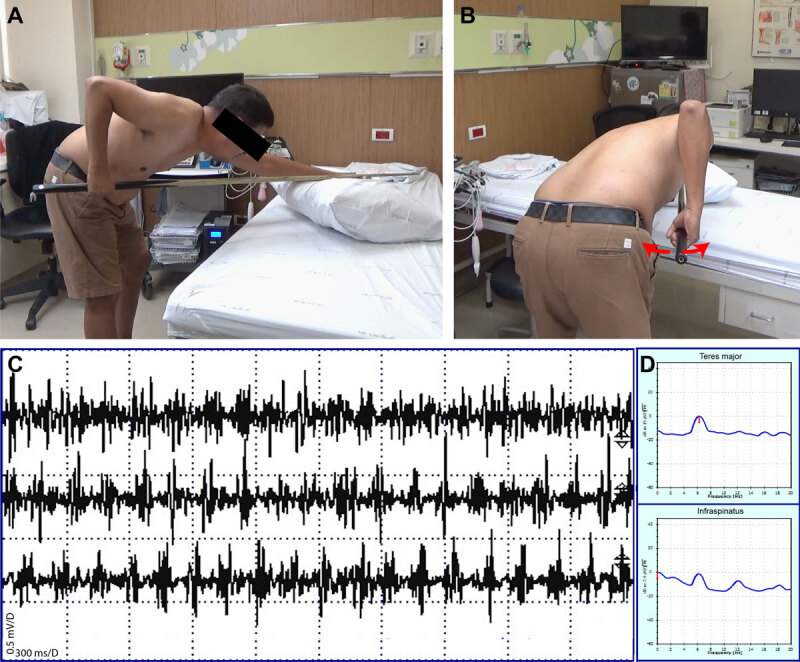
The position that triggered the primary cueing tremor in Case 1. **(A)** View from the right side of the patient. **(B)** View from the posterior aspect. Surface electromyographic studies in Case 1 revealed characteristic features of tremor with regular oscillations **(C)** recording from teres major. Each small square represents a 300-millisecond duration. The spectral peak analysis confirmed a 6.3-Hz frequency in the teres major and infraspinatus muscles **(D)**.

A 55-year-old right-handed professional billiards player presented for evaluation of a right arm tremor while holding a billiards cue. He owned a billiards club and played daily. He noticed the tremor ten years prior to presentation; it had slowly progressed but had stabilized in the past four years. Tremor markedly impaired his performance. He denied locking or movement arrests when moving a cue. There was no tremor when holding a cue with the left hand or performing other activities, including writing. He reported a marked improvement in tremor after drinking 1.5 liters of beer (5.0% alcohol by volume). There was no family history of tremor. On examination, as soon as he held a cue backward with an extended right shoulder (Figure 1A and 1B), there was a prominent arm tremor in the internal-external rotation axis, resulting in a side-to-side tremor of the forearm (Video 1). Once he advanced the cue forward when shooting, the tremor disappeared. Due to this tremor, he sometimes compensated by shooting quickly without a prolonged aiming phase. There was also a very mild postural and intention (i.e., terminal kinetic tremor or tremor that increases in amplitude when approaching a target) of bilateral hands, slightly more significant on the left, of which he was unaware. There were no phenomenologic features of dystonia, including sensory tricks. Surface electromyography (EMG) of the infraspinatus and teres major muscles demonstrated a 6.3-Hz tremor when he held a cue backward with an extension of the right shoulder (Figure 1C and 1D). He was unable to tolerate multiple trials of medications, including propranolol, topiramate, gabapentin, and zonisamide. Primidone was not available in Thailand. Fifty units of onabotulinumtoxinA were injected under EMG and electrical stimulation guidance into the infraspinatus with mild-to-moderate subjective improvement in tremor, but injections were discontinued due to financial concerns.

**Video 1:** This video demonstrates Case 1 and is comprised of Segment 1 and Segment 2, both of which illustrate the same tremor pattern. Segment 2 was recorded with the patient’s upper trunk exposed to better visualize muscle activity. The tremor is best appreciated in the front and back oblique views, where it is more prominent than in the lateral view. The video demonstrates a right arm tremor that occurs specifically when the patient holds the cue in a backward position with an extended right shoulder. The tremor occurs primarily in the internal-external rotation axis, resulting in side-to-side oscillations of the forearm. Of note, the tremor disappears briefly when the patient advances the cue forward to shoot (time mark: 0:40), before reappearing shortly afterward.

### Case 2

A 66-year-old right-handed male professional billiards competitor and instructor was referred for evaluation of tremor. While no other activities were affected, he relied on billiards as a source of income. He had no family history of tremor and denied drinking alcohol. His exam revealed no rest tremor, but there was a fine bilateral postural and terminal kinetic tremor which he had not previously noticed. Likewise, there was no parkinsonism or dystonic features. However, when adopting his shooting stance with a pool cue, he developed a coarse whole hand tremor, most prominently in the left hand, which was acting as the bridge to stabilize the cue. A subtle tremor was also observed in the right hand holding the cue. Notably, the tremor could be recapitulated without the pool cue. There were no alleviating maneuvers. Of note, the video of Case 2 was not available. He could not tolerate propranolol, primidone, gabapentin, or topiramate. A trial of transcutaneous alternating patterned stimulation (TAPS) was unsuccessful. A modest dose of clonazepam was effective in treating the tremor, but he was limited by sedation. Ultimately, he underwent bilateral deep brain stimulation (DBS) intended for the ventralis intermedius (Vim) nucleus of the thalamus, but the electrodes were instead placed into the subthalamic nucleus (STN). Postoperative imaging and subsequent programming revealed that the electrodes were placed more lateral and deeper than intended, resulting in current spread involving the STN. This was also supported by stimulation-induced dyskinesias in some stimulation settings. Despite this, the patient attained a subjective 70% improvement in tremor, enough to play in smaller tournaments and continue to teach. The benefit had been sustained for over 24 months as of the most recent follow-up. The most recent stimulation parameters, using directional leads from the Boston Scientific Vercise Genus R16 system, were as follows. For the right Vim, stimulation was delivered at an amplitude of 4.0 milliamps (mA), with a pulse width of 60 microseconds (μs) and a frequency of 130 Hertz (Hz). The electrode configuration and directional current distribution were as follows: contact 3B+ 20%, 2B+ 80%, 3C– 3%, 3A- 2%, 2C- 91%, and 2A- 4%. For the left Vim, the parameters were an amplitude of 4.2 mA, pulse width of 60 μs, and frequency of 130 Hz. The directional settings included Case+ 40%, contact 4+ 20%, 3C+ 19%, 3A+ 16%, 2C+ 5%, 3B- 80%, and 2B- 20%.

## Discussion and literature review

Here we report two cases of TST when playing billiards. Both patients also demonstrate position-specific tremor: in Case 1, the tremor occurred specifically when holding the cue with right shoulder extension when aiming for the ball, and in Case 2, the more dramatic tremor was when adopting the bridge to stabilize the cue. Thus, this can also be called “primary cueing tremor”, in analogy to other TSTs such as primary writing tremor or primary bowing tremor. Although termed task-specific tremor, the tremor may occur either when performing the task itself or when assuming a posture closely associated with the task, even in the absence of object involved (e.g., without holding the cue). This latter phenomenon in which tremor is elicited by task-related positioning is referred to as position specificity and is recognized in other forms of TST, such as primary writing tremor [5,11].

Task-specific movement disorders in athletes have been reported, with most cases being task-specific dystonia. Based on a literature review, only two cases of task-specific dystonia in billiards players have been documented (Table 1) [7,10]. Both professional players experienced dystonia in the dominant arm when positioning and holding the cue to prepare for striking the ball with the right arm flexed and the elbow behind the trunk, while the left arm was extended, a position similar to our cases but with different phenomenology. The patients described a “locked” or “stiff” sensation in the right arm. One case demonstrated electrophysiologic evidence of co-contraction of the triceps and deltoids when pulling the cue backward, which disappeared when the cue was not held. One player reported no alleviating maneuvers, while the other was able to overcome by focusing his sight on the afflicted arm.

**Table 1:** Literature review of phenomenology and treatments of billiards-related movement disorders compared to the novel cases.


REFERENCE	SEX	AGE	AGE AT ONSET	DURATION OF PLAYING (YEARS)	PHENO MENOLOGY	AFFECTED BODY REGION	SYMPTOM DESCRIPTION	POSITION SPECIFICITY	ELECTROPHYSIOLOGIC FINDINGS	TREATMENT TRIAL	TREATMENT RESPONSE*

Case 1	Male	55	51	20	Tremor	Dominant arm (right)	Tremor when holding a cue backward	Yes, when holding a cue with extension of the right shoulder	Regular oscillations (6.3 Hz) recording from the right teres major and infraspinatus muscles	Propranolol, topiramate, gabapentin, zonisamide, BoNT	Poor to medications; mild-to-moderate to BoNT

Case 2	Male	66	56	N/A	Tremor	Mainly in non-dominant arm (left), subtle in dominant arm (right)	Tremor when adopting his shooting stance with a pool cue	Yes, when the left hand was acting as the bridge	N/A	Propranolol, topiramate, gabapentin, primidone, clonazepam, TAPS, Vim DBS	Poor to medications and TAPS; moderate to DBS

Smilowska et al [10]	Male	57	52	N/A	Dystonia	Dominant arm (right)	Arm ‘blocked’ when hitting the ball	Yes, when moving the arm back and forth in a pendulum motion, prior to hitting the white cue ball	Co-contraction of right triceps brachii and biceps brachii and excessive activity in the right deltoid and right brachioradial muscles while holding a billiards stick.	BoNT	Excellent

Lee et al [7]	Male	52	47	30	Dystonia	Dominant arm (right)	Arm being ‘locked’ when he tried to strike a ball	Yes, when extending the elbow	Co-contraction of triceps and deltoids immediately after bicep contraction when pulling the cue backward	Propranolol, benzodiazepine, anticholinergics, BoNT	Poor

Salari et al [9]	Male	34	32	22	“Tremor”	Head	Involuntary neck movements with posture in the position of striking a ball	Yes, when bending his trunk at the hips at approximately 80–90° while supporting his head against gravity by keeping his neck straight	N/A	N/A	N/A


*Treatment response refers to the outcome reported in each individual patient, as described in the original case report. These descriptors reflect subjective or observed clinical effects and are not intended to imply group-level generalizations. As more cases and objective response measures become available, broader conclusions may be possible. Abbreviations: BoNT = botulinum toxin; DBS = deep brain stimulation; TAPS = transcutaneous alternating patterned stimulation; Vim, ventral intermediate nucleus of the thalamus, N/A, not applicable.

There is also one report of position-specific tremor in a billiards player involving the head alone. This patient experienced a head tremor only when attempting to hit the ball by bending the trunk at the hips at an 80–90 degree angle. This tremor was described as jerky, high-frequency, and low-amplitude. The tremor did not occur in other positions; however, the tremor also emerged when bending forward without holding the cue. This report did not clarify whether this movement was a “tremor” or “dystonic tremor,” but the authors suggested it might represent task-specific cervical dystonia.

Distinguishing the phenomenology of billiards-related movement disorders is crucial (Table 2), as this implicates different treatment approaches. In dystonia, abnormal posturing may be observed, with patients reporting symptoms of freezing or locking that impair control of the affected arm. In contrast, tremor is characterized by regular oscillations around an axis. Position specificity can also be seen in both dystonia and tremor, as in our cases. Electrophysiologic studies can help distinguish tremor from dystonia: there are regular frequencies in the former, whereas there is co-contraction of agonist and antagonist muscles in the latter. The presence of 6-Hz tremor, subtle terminal kinetic and postural tremor, as well as alcohol responsivity in Case 1, suggests that primary cueing tremor could represent a variant of ET, as proposed in other TSTs. Importantly, the distinction between tremor and dystonia has direct therapeutic implications. Pharmacological treatments, preferred DBS targets (e.g., Vim for tremor vs. GPi or ventral oralis [Vo] complex for dystonia) [12,13], and botulinum toxin injection strategies differ substantially. For example, tremor is often managed by injecting both agonist and antagonist muscles to dampen oscillations [14], whereas dystonia typically requires targeting primary dystonic muscles. Accurate classification is also increasingly relevant as new symptomatic therapies become available, potentially tailored to specific movement disorder phenomenology.

**Table 2:** Differentiating between primary cueing tremor from billiards-related dystonia.


	PRIMARY CUEING TREMOR	BILLIARDS-RELATED DYSTONIA

Symptoms	Shaking of the arm while cueing	Sudden pause, characterized by “locking” or “freezing” of the dominant arm while cueing; dystonic jerking movements have not been reported

Phenomenology	Regular oscillations of the movements	Abnormal posturing while cueing

Position specificity	Yes	Yes

Involved muscles	Infraspinatus and teres major (in case of tremor in internal-external rotation axis of the arm)	Triceps, biceps, brachioradialis, deltoid

Electrophysiology	Regular oscillations with approximately 6-Hz frequency	Co-contraction of agonists and antagonists

Alcohol responsivity	Yes	No

Response to pharmacological treatment	Poor	Poor

Response to botulinum toxin	Varying from poor to mild-to-moderate	Excellent

Proposed deep brain stimulation target	Ventral intermediate nucleus (Vim) of the thalamus	Ventralis oralis anterior (Voa) and posterior (Vop) of the thalamus or globus pallidus interna (GPi)


Pharmacological trials provide unsatisfactory results, though this is not uncommon. Case 1 had mild-to-moderate improvement with botulinum toxin injections, but he did not want to continue due to financial concerns. Case 2 similarly showed subtle action tremor and had significant, albeit functionally suboptimal, improvement with DBS. The outcome may be considered “suboptimal” in the context of the high precision and performance demands required of a professional billiards player. In other case reports of billiards-related dystonia, one patient had an excellent response to botulinum toxin injections, while others did not.

In Case 2, the benefit observed with DBS is noteworthy, particularly given evidence of stimulation-induced dyskinesias, which support stimulation of the subthalamic nucleus (STN). We believe the clinical benefit likely resulted from activation of the STN and adjacent structures within tremor circuits. Although STN stimulation is typically used in Parkinson’s disease, it is known to have non-specific anti-tremor effects even when not directly targeted. Furthermore, stimulation of the zona incerta (ZI), located dorsal to the STN, and nearby components of the dentato-rubro-thalamic tract (DRTT) within the posterior subthalamic area (PSA) has been shown to suppress tremor [15]. The PSA, which encompasses the ZI and the prelemniscal radiations, comprising both DRTT and pallidothalamic fibers [16,17], play a key role in tremor modulation and may require lower stimulation thresholds than the Vim [18].

Our two cases expand the phenomenological spectrum of task-specific movement disorders in billiards players to include tremor affecting the arm, in addition to billiards-related dystonia. More reported cases in the future can provide additional insights regarding phenomenological features, treatment responses, and trials such as sodium oxybate, given alcohol responsivity, natural history, and possible pathophysiology in primary cueing tremor. Recognizing this entity and distinguishing it from other phenomenology will help provide appropriate information regarding natural history and treatment in billiards players, as these movements can significantly impact their performance and professional abilities.

## Conclusion

Primary cueing tremor is a task-specific movement disorder that can be found in billiards players, in addition to billiards-related dystonia. This expands the phenomenology of billiards-related movement disorders. Our findings suggest that primary cueing tremor may represent a variant of ET. Recognizing primary cueing tremor may have implications for treatment, natural history, and pathophysiology, which likely differ from those of billiards-related dystonia.
